# Energy-Aware and Secure Task Offloading for Multi-Tier Edge-Cloud Computing Systems

**DOI:** 10.3390/s23063254

**Published:** 2023-03-20

**Authors:** Hatem A. Alharbi, Mohammad Aldossary, Jaber Almutairi, Ibrahim A. Elgendy

**Affiliations:** 1 Department of Computer Engineering, College of Computer Science and Engineering, Taibah University, Al-Madinah 42353, Saudi Arabia; 2 Department of Computer Science, College of Arts and Science, Prince Sattam Bin Abdulaziz University, Al-Kharj 16278, Saudi Arabia; 3 Department of Computer Science, College of Computer Science and Engineering, Taibah University, Al-Madinah 42353, Saudi Arabia; 4 Department of Computer Science, Faculty of Computers and Information, Menoufia University, Shibin El Kom 32511, Egypt

**Keywords:** mobile edge computing, task offloading, security, unmanned aerial vehicle, compression

## Abstract

Nowadays, Unmanned Aerial Vehicle (UAV) devices and their services and applications are gaining popularity and attracting considerable attention in different fields of our daily life. Nevertheless, most of these applications and services require more powerful computational resources and energy, and their limited battery capacity and processing power make it difficult to run them on a single device. Edge-Cloud Computing (ECC) is emerging as a new paradigm to cope with the challenges of these applications, which moves computing resources to the edge of the network and remote cloud, thereby alleviating the overhead through task offloading. Even though ECC offers substantial benefits for these devices, the limited bandwidth condition in the case of simultaneous offloading via the same channel with increasing data transmission of these applications has not been adequately addressed. Moreover, protecting the data through transmission remains a significant concern that still needs to be addressed. Therefore, in this paper, to bypass the limited bandwidth and address the potential security threats challenge, a new compression, security, and energy-aware task offloading framework is proposed for the ECC system environment. Specifically, we first introduce an efficient layer of compression to smartly reduce the transmission data over the channel. In addition, to address the security issue, a new layer of security based on an Advanced Encryption Standard (AES) cryptographic technique is presented to protect offloaded and sensitive data from different vulnerabilities. Subsequently, task offloading, data compression, and security are jointly formulated as a mixed integer problem whose objective is to reduce the overall energy of the system under latency constraints. Finally, simulation results reveal that our model is scalable and can cause a significant reduction in energy consumption (i.e., 19%, 18%, 21%, 14.5%, 13.1% and 12%) with respect to other benchmarks (i.e., local, edge, cloud and further benchmark models).

## 1. Introduction

Recently, Unmanned Aerial Vehicles (UAVs) have been gaining popularity and attracting considerable attention, where they can offer numerous types of applications and services including augmented and virtual reality, the Internet of Vehicles (IoV), video and 3D games, efficient manufacturing inspection and e-health [1]. However, the computation capabilities and live battery of UAVs still limit these types of applications from being efficiently processed, where they demand more energy, computation, and storage resources [2].

Consequently, the task offloading concept is first introduced as a potential solution for addressing the limitations of UAVs, where the intensive applications can be remotely processed at a cloud server [3,4]. Despite the advantages of cloud server execution, it is primarily challenged by high-latency and security concerns [5,6]. Subsequently, an edge computing paradigm emerged as a prominent solution to cope with the limitations of cloud through moving the computation and storage capabilities of cloud at the edge of the network, thereby reducing the latency [7,8].

Several models and methodologies for task offloading have been proposed and developed in recent years to address the limitations of these UAV devices with various goals and objectives [9,10]. For example, Sacco et al. [11] proposed a task offloading decision-based distributed algorithm for UAV networks with the goal of improving task completion times while reducing total energy consumption. However, in the Ref. [12], a novel UAV-aided mobile edge computing system is proposed where IoT devices, edge-cloud servers, and UAVs are jointly interacted to address the offer of edge computing for IoT devices with the aim of optimizing the service delay of IoT devices and energy of UAV. Moreover, an energy-aware model is introduced for multi-UAV-aided edge-cloud networks, where UAVs are efficiently cooperated to cover the high-density areas of the network with the aim of optimizing the energy consumption [13]. Further, the UAV-aided edge computing approach is presented in the Ref. [14] to address the task offloading problem among UAV and IoT mobile devices and optimize the amount of consumed energy for completing the applications’ tasks. Xu et al. [15] investigate UAV-aided task offloading model for IoT devices in a smart environment, in which they formulate the task offloading decision for IoT devices as an optimization problem with the aim of optimizing long-term energy consumption. In addition, they proposed a deep reinforcement learning-based algorithm to solve this problem and handle the large-scale environment.

In spite of the fact that there have been recent studies that have focused on task offloading, some of these models are still vulnerable to a variety of attacks, while other frameworks did not take into account how to safeguard the confidential information of users. Moreover, with the advantages of the ECC, only the limited bandwidth condition in the case of simultaneous offloading via the same channel with increasing data transmission of these applications is a critical issue that has not been adequately addressed. Therefore, in this study, an energy-aware task offloading framework is proposed for the ECC system environment, in which we added two layers of compression and security to respectively address the limited bandwidth and security issues. The main contributions reported in this paper are summarized as follows:

- An integer nonlinear optimization problem incorporating task offloading, resource allocation, compression, and security is formulated in order to optimize energy consumption under delay constraints for multi-UAV ECC systems.
- An AES-based technique is introduced as a security layer to protect the data of UAVs’ applications from cyber-attack before they are sent to ECC servers.
- A JPEG compression algorithm is utilized as the most efficient approach to reduce the size of the offloading computation task’s images in the low bandwidth state, thereby improving the performance of the whole system.
- A multi-UAV task offloading algorithm is designed to clearly describe the comprehensive process for deducing the offloading decision for UAVs’ tasks in a systematic way.
- Simulation results revealed that our model is scalable and can save a significant reduction in energy consumption with respect to other benchmark solutions (i.e., local, edge, cloud, and various traditional models).

The rest of this paper is structured as follows. The related work on task offloading strategies is covered in Section 2, and Section 3 presents an introduction to the proposed system model and the formulation of our optimization problem. Then, in Section 4, we provide the solution to our optimization problem and develop an energy-aware and secure task offloading algorithm to derive the solution. In Section 5, simulation-based tests are presented. Finally, the conclusion and future directions are covered in Section 6.

## 2. Related Work

Recently, several models and approaches for task offloading have been developed for UAV-edge-cloud networks to handle the limitations of UAVs with various goals and objectives. Some of these approaches address a single tier with single and multiple-user environments. However, other approaches deal with multi-tiers with single and multiple-user environments. This section provides an overview of common methods and highlights their main objectives and goals.

Wang et al. [16] proposed an agent-enabled task offloading framework for UAV-enabled mobile edge computing systems, in which the UAV, edge and cloud can be integrated to assist the mobile users in processing their tasks on the basis of the proactive interaction and autonomous decision-making features. In addition, they formulate the task offloading delay and task offloading energy to lead the agent for selecting the best offloading plan. However, Li et al. [17] utilized the UAV as an edge server to offer the computational capabilities for IoT devices with the goal of maximizing tasks’ offloading throughput. In addition, a semi-Markov decision process-equivalent form is obtained, and a deep reinforcement learning-based technique is utilized to solve it. In their contribution, Chen et al. [18] proposed an intelligent and efficient task offloading algorithm for UAV-enabled edge computing systems, where the action of offloading can smartly be determined on the basis of a deep Monte Carlo Tree Search algorithm.

Elsewhere, the Refs. [12,19] introduced an offloading model for UAV-assisted edge-enabled IoT networks. Specifically, in the Ref. [19], the UAVs act as small cloudlets for processing the IoT devices’ tasks. In addition, the offloading problem is formulated as an integer optimization with the goal of optimizing energy while fulfilling the quality of services. Then, an effective capacitance offloading game is designed to solve this problem and reach to Nash Equilibrium. On their part, Yu et al. [12] utilized the UAVs to offer computational capabilities for IoT devices in the areas where terrestrial signal obstruction or shadowing prevents IoT devices from connecting to the edge servers. Additionally, the allocation of computing and communication resources, task offloading, and the position of UAVs are jointly formulated as optimization problems with the aim of reducing the service delay and energy of IoT devices. Moreover, the successive convex approximation-based algorithm is developed to solve this problem and derive sub-optimal solutions.

Similar to the highlighted efforts, Sun et al. [20] proposed a new model to optimize the deployment of a multi-UAV for UAV-aided mobile edge computing. In addition, the horizontal and vertical position of UAVs is considered via an integer nonlinear problem with the aim of reducing task completion time. However, Huang et al. [21] and Alhelaly et al. [13] have recently addressed the task offloading problem for IoT devices using UAV-aided fog-enabled IoT networks. Specifically, in the Ref. [21], UAVs are utilized to offer task offloading opportunities for IoT devices through two main links, device-to-device link and ground-to-air link. In addition, transmission power, UAV movement and task offloading are jointly optimized in a problem formulation with the goal of decreasing the overall network overhead. Moreover, a UAV-aided task offloading algorithm is developed to derive the solution efficiently. In the meantime, task offloading and resource allocation are optimized in the Ref. [13] for multi-UAV-aided edge cloud computing networks, where UAVs are deployed as networks to cover high-density and dead areas.

The analysis of the above review provides the following conclusions. Task offloading in multi-UAV ECC systems has been extensively researched and implemented for a variety of applications. However, only a few of these studies take into account multi-edge servers’ systems, whereas the majority of them concentrate on single-edge server systems with or without a cloud node. In addition, most of the current approaches are still vulnerable to a variety of attacks, while other frameworks did not take into account how to safeguard the confidential information of users. Moreover, with the advantages of the ECC, only the limited bandwidth condition in the case of simultaneous offloading via the same channel with increasing data transmission of these applications is a critical issue that has not been adequately addressed. Motivated by these considerations and distinct from the above works, in this study, an energy-aware task offloading framework is proposed for the ECC system environment, in which we introduce two layers of compression and security to respectively address the limited bandwidth and security issues.

## 3. System Model and Problem Formulation

This section starts by introducing a multi-UAV, with a multi-tier ECC system model. Following this, our optimization problem pertaining to task offloading, security, and compression models is formulated with the aim of minimizing energy consumption under delay constraints.

### 3.1. Model of Network

**Figure 1:**
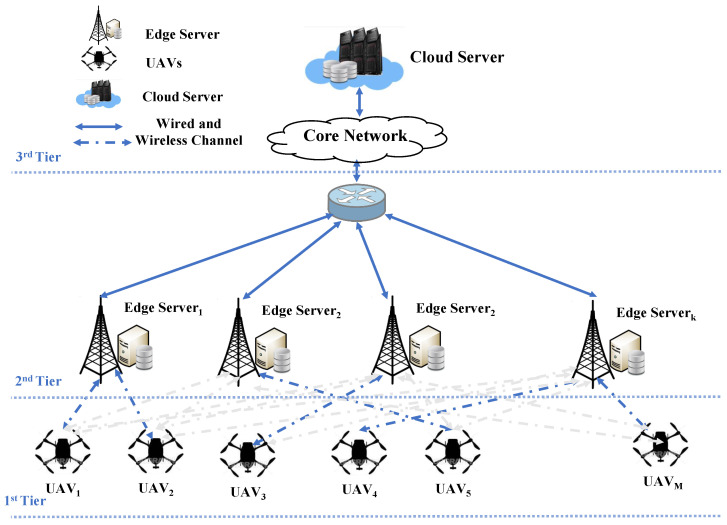
System model.

This subsection presents the proposed system model, where multi-UAVs with a multi-tier ECC environment are considered. Our objective is to reduce the system’s energy with latency constraints for a multi-tier system. This system is composed of three layers, shown in Figure 1, where we have a M set of UAVs connected with k set of edge servers. In addition, each UAV has a N set of intensive-tasks that should be completed. Moreover, the edge servers’ sets are managed through a centralized router which controls the traffic and addresses the load among them as well as links them with a single remote cloud. In this paper, M={1,2,…,M}, N={1,2,…,N}, and K={0,1,2,…,K+1} denote the sets of UAVs, tasks and available servers for task execution, where 0 indicates that the computation tasks will be executed locally at UAV’s resources and K+1 indicates that computation tasks will be executed remotely at the cloud node. More specifically, if the value of k is set to zero, this means the computation task will be performed locally at the device. Meanwhile, if the value of *k* is equal to K+1, this means that the computation task will be performed at the cloud node. Otherwise, the computation task will be performed at one of the available edge servers depending on the value of *k*. Through wireless channels, UAVs are also able to obtain access to the resources of edge servers in order to offload some of the computation tasks for execution.

Let $\alpha_{ijk}\in \left\{0,1\right\}$ denote the offloading decision for the task *j* of UAV *i* that will be executed on server *k*. More specifically, ($\alpha_{ij0}$ = 1) denotes local execution, ($a_{ijK+1}=1$) denotes cloud execution and ($\alpha_{ijk}=1,\forall k\in \left[1..K\right]$) denotes edge server execution. Moreover, each task must be completed on only one server, wherever local, edge, or cloud. Therefore, we can guarantee these requirements through the following equation:

(1) $$\sum_{k=0}^{K+1}\alpha_{ijk}=1$$

where 0 and k+1 indicate the local and cloud execution and k∈[1,..,k] indicates the edge server, where at least one of them must be satisfied as follows:

(2) $$\alpha =\left\{\begin{array}{cc} \alpha_{i,j,0}=1 & Local\;Execution\\ \sum_{k=1}^{K}\alpha_{i,j,k}=1 & Edge\;Execution\\ \alpha_{i,j,K+1}=1 & Cloud\;Execution \end{array}\right.$$

### 3.2. Model of Communication

This subsection presents the communication model for our environment, where it has a M set of UAVs connected with k set of edge servers. In addition, each UAV has a N set of intensive-tasks that should be completed. These tasks can be represented using quadruple values $\left\{a_{ij},b_{ij},c_{ij},d_{ij}\right\}$, in which $a_{ij}$, $b_{ij}$, $c_{ij}$ and $d_{ij}$ indicate the input size, output size, CPU cycles and required deadline for each task *j* of device *i* that can be given via task execution and careful profiling [22,23]. Based on the work in the Ref. [24], we decided to disregard the consumption overhead for sending the result back to UAVs, bij, because the output data for the task were small compared with the input data.

This study considers orthogonal frequency division multiple access methods utilized for simultaneous offloading in the same channel to alleviate the intracellular interference in the up-link transmission [25]. Consequently, the available transmission and receiving data rate for each UAV can be defined regrinding the Shannon law as follows:

(3) $$R_{ik}=B_{ik}\log_{2}\left(1+\frac{p_{i}^{T}G^{2}}{\omega B_{ik}}\right)$$

where $B_{ik}$, and $p_{i}^{T}$ denote the up-link bandwidth and transmission power of UAV, respectively. In addition, *G* and ω indicate the channel gain and noise power of the edge server.

### 3.3. Model of Computation

This section presents the computation model for our environment, where it has a M set of UAVs connected with a k set of edge servers. In addition, each UAV has a N set of intensive tasks that should be completed. Consequently, the amount of time required for calculation in both local and remote execution is demonstrated in further depth in the following subsections.

#### 3.3.1. Local Execution

In this study, we take into account the fact that various UAVs may have varying levels of processing capability. Therefore, regarding local execution, the amount of time it takes and the amount of energy needed to do all the computations locally on each UAV *i* can be calculated regarding Equations (4) and (5):

(4) $$T_{ij}^{L}=\frac{c_{ij}}{f_{i}^{L}}$$

(5) $$E_{ij}^{L}=\psi_{i}c_{ij}$$

where $\psi_{i}$ and $f_{i}^{L}$ denote the energy consumed per cycle of the CPU and UAV *i* computing capability.

#### 3.3.2. Remote Execution

This subsection introduces the remote execution for UAV’s tasks, in which each task will be allocated and then executed at one of the available servers. Subsequently, the amount of time it takes to do all the computations remotely at one of the servers, edge or cloud, can be respectively calculated regarding Equations (6) and (7):

(6) $$T_{ij}^{e}=T_{ij}^{off}+T_{ij}^{e\_ex}$$

(7) $$T_{ij}^{e}=T_{ij}^{off}+\Delta +T_{ij}^{c\_ex}$$

where Δ denotes the propagation delay between the edge server and cloud as well as $=T_{ij}^{off}$, $T_{ij}^{e\_ex}$, and $T_{ij}^{c\_ex}$ indicate the offloading, edge execution, and cloud execution, respectively, which can be expressed as:

(8) $$T_{ij}^{off}=\frac{a_{ij}}{R_{ik}}$$

(9) $$T_{ij}^{e\_ex}=\frac{c_{ij}}{f_{i}^{e}}$$

(10) $$T_{ij}^{c\_ex}=\frac{c_{ij}}{f_{i}^{c}}$$

where $f_{i}^{c}$ and $f_{i}^{c}$ denote the assigned capabilities for device *i* at the edge and cloud servers.

Moreover, the amount of energy needed to do all the computations remotely at one of the servers can be calculated regarding Equation (11):

(11) $$E_{ij}^{R}=p_{i}T_{ij}^{off}$$

Note that the computing resources of edge servers are denoted by $F_{k}$ which will be allocated to all the devices that are connected through the offloading process and thereby should be limited according to the following equation:

(12) $$\sum_{i=1}^{M}\sum_{i=1}^{N}\sum_{i=1}^{K}\alpha_{ijk}f_{i}^{e}\leq F_{K}$$

### 3.4. Model of Security

In the ECC paradigm, each U, such as AV, transmits data from the application to the ECC server over a wireless channel. Private information such as images, health records, or transactions may be included in this data. However, recently, it has been proven that this channel is vulnerable to penetration and the mobile user is susceptible to impersonation attacks in mobile edge computing [26]. Moreover, offloading tasks to MEC servers are still vulnerable to external security threats (e.g., snooping, eavesdropping and alteration). As a result, without adequate security procedures, the advantages of the ECC paradigm will be swiftly outweighed by the harm that hostile adversaries and cyberattacks will do. To avoid these threats, it is necessary to encrypt the transferred data and shield it from cyberattacks with an effective and secure layer. Therefore, in this study, we suggest a conventional Advanced Encryption Standard (AES) cryptographic approach strengthened by the Genetic Algorithm (GA), an evolutionary algorithm to efficiently protect the data [27].

AES is a symmetric encryption method that has been shown to be effective in a variety of applications. In addition, AES is selected as a cryptographic approach due to its standardization and high performance. Nevertheless, due to the straightforward and fundamental nature of its algebraic structure as well as the use of a single replication method to encrypt each block of the signal, these systems include flaws that make them susceptible to a wide variety of assaults. In addition, the security key is sometimes referred to as the key factor, since it is the component that contributes significantly to the robustness of the algorithm and is considered the main important part of the cryptographic approach. Therefore, GA is utilized with the help of the random number generator method to generate an efficient and more complex key that is difficult to guess or attack. This layer can lessen the complexity of time while defending against various threats.

Subsequently, let βij denote the decision of security for task *j* of UAV *i*, which indicates whether the UAV will protect the transferred data by applying our security layer or not. Specifically, $\beta_{ij}=0$ indicates the data will be transmitted without passing through the layer of security, whereas $\beta_{ij}=1$ indicates the data are sensitive and should go through the layer of security for protecting against cyberattacks. Consequently, as the security decision depends on the privacy requirements of the transmitted data, it will be selected randomly in our simulation.

Finally, the additional time and energy required to conduct the tasks remotely on edge or cloud servers, taking into account the security layer, may be determined as follows:

(13) $$t_{ij}^{enc+dec}=\frac{enc_{ij}}{f_{i}^{l}}+\frac{dec_{ij}}{f_{i}^{r}}$$

(14) $$e_{ij}^{enc}=\psi_{i}enc_{ij}$$

where $enc_{ij}$ and $dec_{ij}$ denote the cycles of CPU for encrypting and encrypting the transmitted data of task *j* locally at UAV *i* and at the edge or cloud server, respectively [28,29].

### 3.5. Model of Compression

The resources for offloading computations could be shared by numerous UAVs in ECC systems. Moreover, with the increasing number of UAVs, the communication bandwidth available for each UAV will decrease, thereby decreasing overall performance. Further, most of the current applications produce a growing amount of data, which will need to be communicated over wireless channels. Consequently, motivated by these considerations, we introduce a new layer of compression to compress the transmitted data before offloading and thereby reduce the communication overhead. More specifically, before offloading to the ECC server, the images and videos from the UAVs’ tasks are respectively compressed using the JPEG and MPEG4 algorithms, which are the most popular and widely used compression algorithms due to their efficiency in terms of compression ratio and processing time [30].

Subsequently, let γij denote the decision of compression for task *j* of UAV *i*, which indicates whether the UAV will compress the transferred data by applying our compression layer or not. Specifically, $\gamma_{ij}=0$ indicates the data will be transmitted without passing through the layer of compression due to the small size of the network being underloaded. However, $\gamma_{ij}=1$ indicates the data are large and the network is overloaded (i.e., low bandwidth condition) and should go through the layer of compression for reducing the transferred data and thereby improve the overall performance.

Finally, the additional time and energy required to conduct the tasks remotely on an edge or cloud server, taking into account the compression layer, may be determined as follows:

(15) $$t_{ij}^{co+deco}=\frac{co_{ij}}{f_{i}^{l}}+\frac{deco_{ij}}{f_{i}^{r}}$$

(16) $$e_{ij}^{co}=\psi_{i}co_{ij}$$

where $co_{ij}$ and $deco_{ij}$ denote the cycles of CPU for compressing and decompressing the transmitted data of task *j* locally at UAV *i* and at the edge or cloud server, respectively [31].

Furthermore, the transmitted data size is reduced after going through the compression layer. Subsequently, in this study, we adapt the compression ratio’s value to 50, which can reduce the data and at the same time achieve good quality [31,32], and the data are denoted as $a_{ij}^{co}$. Therefore, the time $T_{ij}$ and energy $E_{ij}$ after applying compression can be determined as follows:

(17) $$T_{ij}^{off\_co}=\frac{a_{ij}^{co}}{R_{ik}}$$

(18) $$E_{ij}^{R\_co}=p_{i}T_{ij}^{off\_co}$$

Additionally, considering the models mentioned above (i.e., communication, computation, security, and compression), the overhead time $T_{ij}$ and energy $E_{ij}$ can be determined as follows:

(19) $$\begin{array}{cc} T_{ij} & =[\alpha_{ij0}T_{ij}^{L}+\sum_{k=1}^{K}\alpha_{ijk}[\gamma_{ij}\left(t_{ij}^{co+deco}+\left(\beta_{ij}\left(t_{ij}^{enc+dec}\right)\right)+T_{ij}^{off\_co}+T_{ij}^{e\_ex}\right)+\\ \; & \left(1-\gamma_{ij}\right)\left(\left(\beta_{ij}\left(t_{ij}^{enc+dec}\right)\right)+T_{ij}^{off}+T_{ij}^{e\_ex}\right)]\\ \; & \alpha_{ijk+1}[\gamma_{ij}\left(t_{ij}^{co+deco}+\left(\beta_{ij}\left(t_{ij}^{enc+dec}\right)\right)+T_{ij}^{off\_co}+\Delta +T_{ij}^{c\_ex}\right)+\\ \; & \left(1-\gamma_{ij}\right)\left(\left(\beta_{ij}\left(t_{ij}^{enc+dec}\right)\right)+T_{ij}^{off}+\Delta +T_{ij}^{c\_ex}\right)]] \end{array}$$

(20) $$\begin{array}{cc} E_{ij} & =[\alpha_{ij0}E_{ij}^{L}+\sum_{k=1}^{K+1}\alpha_{ijk}[\gamma_{ij}\left(E_{ij}^{R\_co}+\left(\beta_{ij}\left(e_{ij}^{enc}\right)\right)\right)+\\ \; & \left(1-\gamma_{ij}\right)\left(E_{ij}^{R}+\left(\beta_{ij}\left(e_{ij}^{enc}\right)\right)\right)]] \end{array}$$

### 3.6. Formulation of Problem

In this section, the formulation of our problem for multi-UAVs with multi-task ECC systems is introduced, where task offloading, computation and communication, security, and compression models are all considered. Consequently, the following problem is our optimization, where minimizing the overhead energy of the system with delay constraints is the main objective:

(21) $$\begin{array}{cccccc} \; & \min_{\alpha ,\beta ,\gamma } & \; & \left[\sum_{i=0}^{M}\sum_{j=0}^{N}E_{ij}\right] & \; & \\ \; & s.t & \; & E_{ij}\leq E_{ij}^{L}, & \; & C1\\ \; & s.t & \; & T_{ij}\leq d_{ij}, & \; & C2\\ \; & \; & & \sum_{k=0}^{K+1}\alpha_{ijk}=1, & \; & C3\\ \; & \; & & \sum_{i=1}^{M}\sum_{i=1}^{N}\sum_{i=1}^{K}\alpha_{ijk}f_{i}^{e}\leq F_{K}, & \; & C4\\ \; & \; & & \alpha_{ijk}\in \left\{0,1\right\}, & \; & C5\\ \; & \; & & \beta_{ij}\in \left\{0,1\right\}, & \; & C6\\ \; & \; & & \gamma_{ij}\in \left\{0,1\right\}, & \; & C7 \end{array}$$

where the first and second constraints (C1 and C2) handle the energy and delay bound and the third constraint C3 guarantees that each task will only be processed once. Moreover, the fourth constraint C4 addresses the capability bound of edge servers and the task offloading, compression and security decisions are guaranteed to be binarized through the last three constraints (C5, C6 and C7).

To solve the optimization problem, it is necessary to determine the optimal value for offloading, compression, and security decisions. However, this problem is classified as NP-Hard and it is difficult to solve it in polynomial time because its variables are binary, and thereby its feasible set is not convex. Moreover, as the three binary variables α, β and γ are multiplied, the objective function is also non-convex [33,34]. Therefore, we transform our optimization problem into a convex one using linearization and relaxation approaches, as shown in the following section.

## 4. Solution of Problem

As mentioned above that our formulated problem is classified as NP-Hard, it is difficult to solve it in polynomial time because its variables are binary and thereby its feasible set is not convex. Moreover, as the three binary variables α, β and γ are multiplied, the objective function is also non-convex. Consequently, we seek to convert this problem into an equivalent convex form that can be effectively addressed and solved. This reformation is composed of two main steps, namely, linearization and relaxation, where the problem is first transformed into a linear one and then the binary variables are relaxed to real variables.

### 4.1. Linearization Transform

In linearization, a nonlinear model is reformulated into a linear one that can be solved more easily. This reformulation can be obtained by adding an auxiliary variable $\delta_{ij}$ to compensate for the multiplication of $\alpha_{ij}$ and $\gamma_{ij}$. Additionally, three inequality constraints will be added to our model, which are:

(22) $$\begin{array}{cccccc} \; & \; & & \delta_{ij}\leq \alpha_{ij}, & \forall i,j & C8\\ \; & \; & & \delta_{ij}\leq \gamma_{ij}, & \forall i,j & C9\\ \; & \; & & \delta_{ij}\leq \alpha_{ij}+\gamma_{ij}-1, & \forall i,j & C10 \end{array}$$

where these constraints ensure that the auxiliary variable δ will be zero in the case of α or γ is 0, and will be one in the case of α and γ are set to 1 [35].

### 4.2. Relaxation Transform

In the relaxation step, the binary variables α, γ and δ are relaxed to real values between zero and one and can be denoted as $\alpha_{ij}\in \left[0,1\right]$, $\gamma_{ij}\in \left[0,1\right]$ and $\delta_{ij}\in \left[0,1\right]$ [36]. Therefore, the equivalent form of our problem after applying the linearization and relaxation approaches can be as follows:

(23) $$\begin{array}{cccccc} \; & \min_{\alpha ,\beta ,\delta ,\gamma } & \; & \left[\sum_{i=0}^{M}\sum_{j=0}^{N}En_{ij}\right] & \; & \\ \; & s.t & \; & En_{ij}\leq E_{ij}^{L}, & \; & C1\\ \; & s.t & \; & Tn_{ij}\leq d_{ij}, & \; & C2\\ \; & \; & & \sum_{k=0}^{K+1}\alpha_{ijk}=1, & \; & C3\\ \; & \; & & \sum_{i=1}^{M}\sum_{i=1}^{N}\sum_{i=1}^{K}\alpha_{ijk}f_{i}^{e}\leq F_{K}, & \; & C4\\ \; & \; & & \alpha_{ijk}\in \left[0,1\right], & \; & C5\\ \; & \; & & \beta_{ij}\in \left[0,1\right], & \; & C6\\ \; & \; & & \gamma_{ij}\in \left[0,1\right], & \; & C7\\ \; & \; & & \delta_{ij}\leq \alpha_{ij}, & \forall i,j & C8\\ \; & \; & & \delta_{ij}\leq \gamma_{ij}, & \forall i,j & C9\\ \; & \; & & \delta_{ij}\leq \alpha_{ij}+\gamma_{ij}-1, & \forall i,j & C10 \end{array}$$

where $En_{ij}$ and $Tn_{ij}$ are the energy and time after compensating with the auxiliary variable.

### 4.3. Energy-Aware and Secure Task Offloading Algorithm

The construction of an Energy-Aware and Secure Task Offloading Algorithm for Multi-Tier ECC systems is presented in this subsection, which includes a step-by-step process for determining the nearly-optimum task offloading decision for each UAV’s task, which is shown in Algorithm 1.

Firstly, each UAV initializes its offloading and compression decision for its tasks with 0, which indicates the computation tasks will be processed locally at the UAV’s resources without compression. Afterward, the computation requirements for each task and local capabilities for each UAV will be gathered at the control manager via the connected edge server. In addition, the data rate at the edge server is shared among UAVs, and the available data rate for each one is calculated (based on the formulation in Equation (3)) and collected at the control manager. Subsequently, the control manager receives the available computational capabilities for each edge server and then calculates the decision values for the offloading and compression variables $\alpha^{*}$ and $\gamma^{*}$ regarding the formulation in Equation (21). Finally, each UAV receives these decisions via the connected servers.

Algorithm 1 describes the task offloading and compression decision-making process. This algorithm has a computational complexity of O(MNK), where *M* represents the number of Unmanned Aerial Vehicles (UAVs), and *N* and *K* represent the number of computation tasks and edge servers, respectively.
**Algorithm 1** Energy-Aware and Secure Task Offloading Algorithm1:**Initialization**: Each task *j* at UAV *i* is initialized with the offloading and compression decision as $\alpha_{ij0}=1$ and $\gamma_{ij}=0$2:**for all** each UAV *i* and at given time slot *t* **do**3: **for all** each task *j* **do**4:  **Send** requirements for each task and UAV’s capabilities to control the manager through the connected edge servers which includes $\left\{a_{ij},c_{ij},d_{ij},\psi_{i},co_{ij},deco_{ij},p_{i}^{T},f_{i}^{L}\right\}$.5: **end for**6:**end for**7:**Send** the available computational capabilities for each edge server and cloud to control manager.8:**Calculate** the rate $R_{i}k$ for each UAV regarding Equation (3).9:**Derive** the values for offloading and compression decisions regarding the formulation in Equation (21).10:**Transmit** these values to UAVs.

## 5. Performance Evaluation and Discussion

This section first provides a concise and precise description of the experimental setup, and then the experimental results and discussion are presented.

### 5.1. Experiment Setup

MATLAB-based simulations and experiments were performed on a PC of the i7-4770 processor with 3.4 GHz frequency, 16 GB RAM, 512 GB of SSD storage and run using Windows 10. A multi-UAV with a multi-tier ECC environment is considered with K = 5 edge servers, M = 100 UAVs, N = 4 independent tasks, and a single cloud node. To account for the heterogeneous computing capabilities of UAVs, the data size and CPU capability for each task and UAVs are uniformly distributed within (0, 10 MB) and 0.5, 0.6, …, 1.0 GHz, respectively. Edge and cloud servers’ capabilities are respectively set to 100 and 500 GHz. Moreover, the computation demands for each task are set to 500 cycles/byte. The security decision is randomly set using uniform distribution in our simulation where the security decision is based on the privacy requirements of the transmitted data. The system bandwidth, transmission power and background noise are set to 20 MHz, 100 mW, and −100 dBm. Furthermore, in order to solve the optimization problem and determine the solution for each task, GAMS language is utilized to implement the model, and MATLAB is utilized as an interface for reading the model parameters [37]. Finally, on the basis of these parameters, our simulation is carried out 50 times and the average values from the results are computed.

### 5.2. Experiment Results and Discussion

#### 5.2.1. Compression Effect

**Figure 2:**
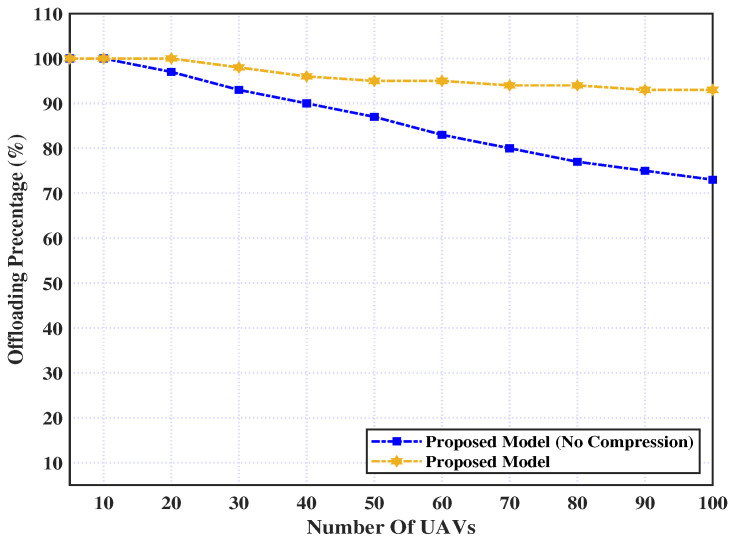
Offloading percentage over number of UAVs.

This subsection illustrates the effect of adding the compression layer to our proposed model, where the model with and without the compression layer is evaluated. Figure 2 shows the percentage of UAVs that execute their tasks remotely with and without the addition of a compression layer. As demonstrated in the graph, the offloading percentage almost reaches 100% for the small number of UAVs (i.e., less than 20) for the two policies. However, for the proposed model without a compression layer addition, this value is steadily decreasing as the number of UAVs increases and reaches 73% for 100 UAVs. However, it slightly decreases after adding the compression layer and reaches 93% for 100 UAVs. This is due to the fact that as the total number of UAVs increases, the communication bandwidth available to each UAV declines, and thereby the proposed model without a compression layer can reject some offloading requests automatically, whereas with the addition of a compression layer, the data are compressed and smartly adapted to the condition of low bandwidth.

**Figure 3:**
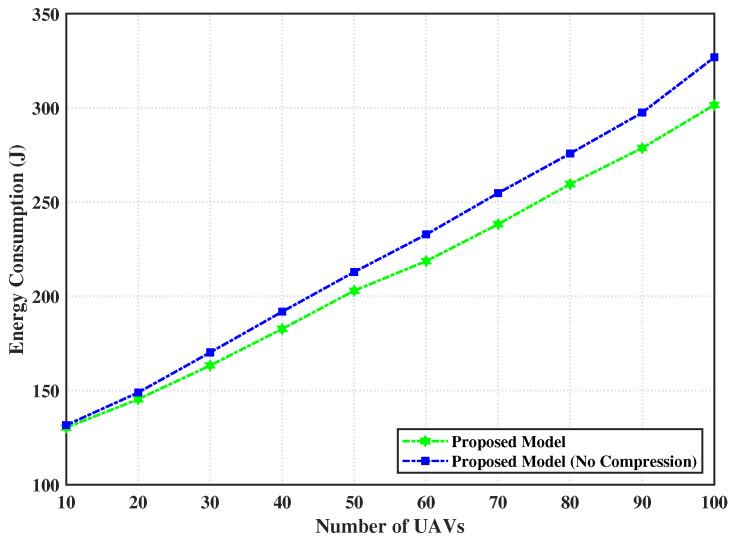
Proposed model with and without compression layer over number of UAVs.

Similarly, Figure 3 shows the consumed energy of executing the tasks for our proposed model with and without the addition of a compression layer relative to the UAV number. From this figure, we observe that the consumed energy of the proposed model by adding a compression layer for a small number of UAVs (i.e., less than 20) is near or equal to the model without a compression layer. However, as the UAVs increase, the gap between the two policies increase, and the proposed model with compression still preserves less consumption of energy with respect to the model without compression. It is attributed to the compression layer having the capability of intelligently adapting to the number of UAVs and thereby reducing overall energy consumption.

#### 5.2.2. Security Effect

**Figure 4:**
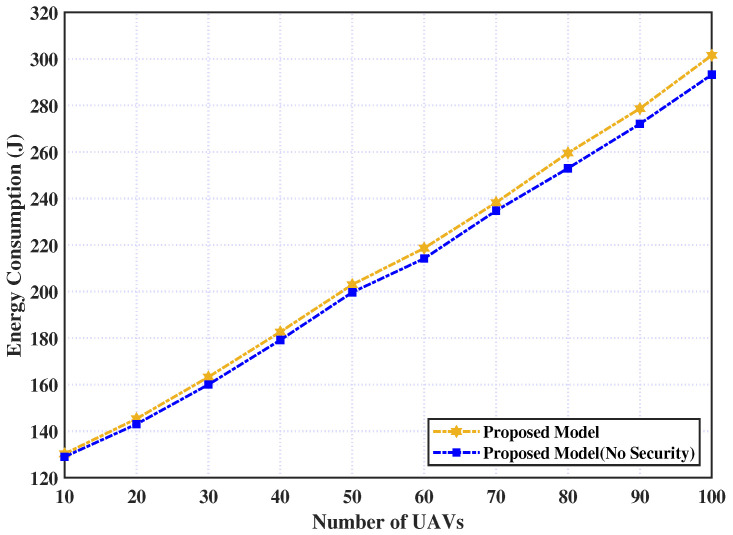
Proposed model with and without the security layer over number of UAVs.

This subsection illustrates the effect of adding the security layer to our proposed model, where the model with and without the security layer is evaluated. The consumed energy of executing the tasks relative to the UAVs’ number is shown in Figure 4. From this figure, we observe that the consumed energy of the proposed model with the security layer addition is larger than the model without security. This is traced to the energy demands associated with the encryption and decryption processes.

#### 5.2.3. Model Performance

This subsection illustrates and verifies the performance of our model, where five different policies are compared:

- **Local Execution Policy:** In this policy, the tasks will be processed using UAV resources without offloading.
- **Edge Execution Policy:** In this policy, the tasks will be executed on the connected edge servers.
- **Cloud Execution Policy:** In this policy, the tasks will be executed at the cloud node.
- **Task Offloading Model [20]:** In this policy, the tasks will be handled via one of the available servers on the basis of work in the Ref. [20].
- **Task Offloading Model [18]:** In this policy, the tasks will be handled via one of the available servers on the basis of work in the Ref. [18].
- **Task Offloading Model [13]:** In this policy, the tasks will be handled via one of the available servers on the basis of work in the Ref. [13].

**Figure 5:**
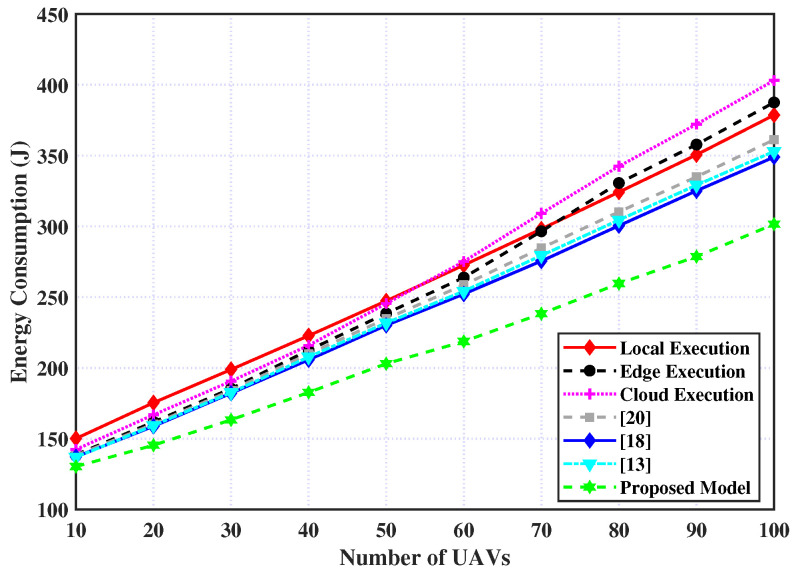
Energy consumption over number of UAVs.

Firstly, the consumed energy for processing the tasks for the six policies relative to different UAVs is shown in Figure 5, in addition to the proposed policy. It is deduced from this figure that energy consumption significantly increases as the number of UAVs increases. However, the proposed model can achieve the best performance in comparison with the other policies with a small number of UAVs, and this gap is increasing with the increasing number of UAVs. Moreover, the cloud and edge policies exceed the local policy with the increasing number of UAVs. This is because the communication channels’ resources have competed among UAVs and consumed more energy during the offloading process, regardless of the offloading policies (i.e., edge, cloud, work in the Refs. [13,18,20], and proposed model). However, the layer of compression has the ability to significantly decrease the consumed energy.

**Figure 6:**
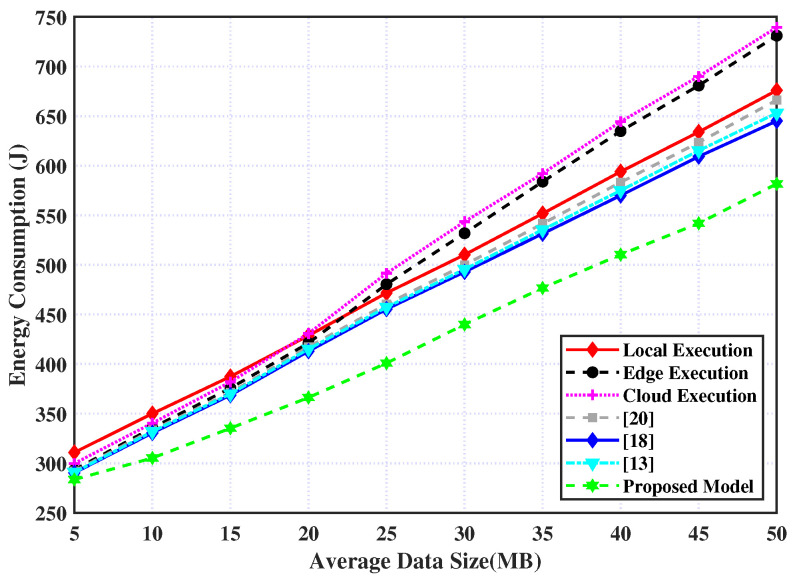
Energy consumption over data size.

Similarly, the consumed energy for processing the tasks for the six policies relative to different data sizes is presented in Figure 6, in addition to the proposed policy. It is observed from this figure that, with a small data size (i.e., less than 20 MB) and the consumed energy for the edge, cloud, and the work in the Refs. [13,18,20], the policies are almost close and less than the local policy, while our model outperforms them. However, as the data size increases, the consumed energy for the edge and cloud policies rapidly increases and exceeds local policy. This is because as data sizes increase, communication times lengthen, which in turn increases energy use. However, our model can be smartly adapted to handle the computation tasks, derive the best decisions, and thereby decrease the consumed energy.

**Figure 7:**
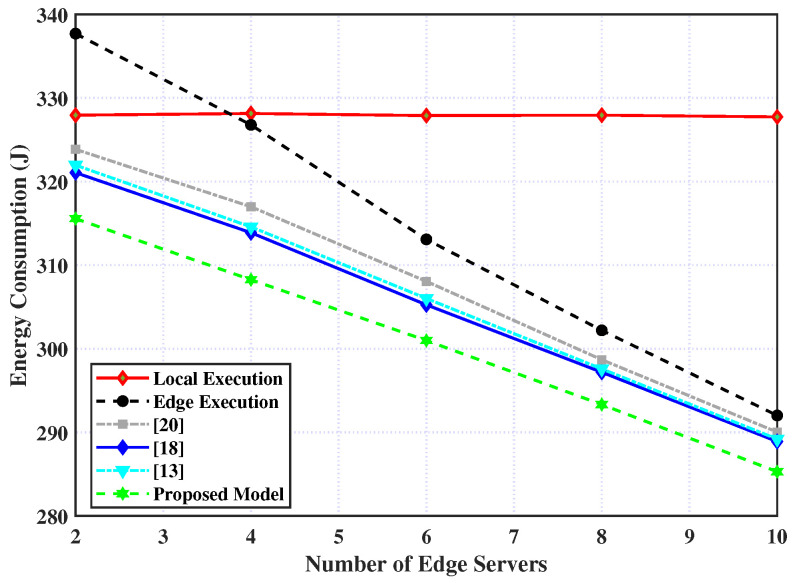
Energy consumption over number of edge servers.

Finally, the consumed energy for processing the tasks for the six policies relative to different numbers of edge servers is presented in Figure 7, in addition to the proposed policy. It is observed from the figure that the local policy is not affected by the number of edge servers while the consumed energy for the other policies steadily decreases with an increase in edge servers. Additionally, the proposed model can outperform the other policies and lower energy consumption. This is attributed to the fact that with the increasing number of edge servers, UAVs are assigned more resources and thereby the consumed energy is decreased, while the edge servers’ resources are not used by the local policy.

## 6. Conclusions

This paper proposed an energy-aware and secure task offloading framework for multi-UAVs with multi-tier ECC systems, where an optimization model of task offloading, computation and communication resources, security, and compression was formulated to minimize overall energy consumption under latency constraints. In addition, a new layer of security was presented to address the potential security threats associated with data transmission. Moreover, the transmitted data can be smartly compressed when the bandwidth is limited and thereby reduce the consumed energy. Our simulation results revealed that our model is scalable and can cause a significant reduction in energy consumption (i.e., 19%, 18%, 21%, 14.5%, 13.1%, and 12%) with respect to other benchmark solutions (i.e., local, edge, cloud, and models in the Refs. [13,18,20]). In ongoing and future work, the UAVs’ mobility will be addressed, where each UAV can dynamically change its location and move between edge servers during the offloading period. Additionally, an intelligent technique based on user behavior will be developed to improve the automated decision of security. Finally, deep learning techniques will be used to handle problem modeling and decisions as well as the complexity of ECC systems. 

## Data Availability

Not applicable.
